# Antecedents and Covariates of Alcohol Consumption among Swiss Male Conscripts

**DOI:** 10.3390/ijerph6030958

**Published:** 2009-03-02

**Authors:** Mario Mueller, Ingo Kipke, Franz Frey, Wulf Rossler, Gianpiero Lupi, Stefan Vetter

**Affiliations:** 1 University of Zurich, Centre for Disaster and Military Psychiatry, Birchstrasse 3, 8057 Zurich, Switzerland; E-mail: stefan.vetter@access.uzh.ch; 2 Institute for Therapy Research, Parzivalstrasse 25, 80804 Munich, Germany; E-mail: kipke@ift.de; 3 Medical Services of the Swiss Armed Forces, Worblentalstrasse 36, 3063 Ittigen, Switzerland; E-mails: Franz.Frey@vtg.admin.ch (F.F.); Gianpiero.Lupi@vtg.admin.ch (G.L.); 4 University of Zurich, Research Unit for Social and Clinical Psychiatry, Militärstrasse 8, 8021 Zurich, Switzerland; E-mail: roessler@dgsp.uzh.ch

**Keywords:** Alcohol consumption, associated factors of alcohol use, abstainers, J-shaped curve, multinomial logistic regression

## Abstract

This study aimed to investigate prevalence and correlates of alcohol consumption frequency in a sample of Swiss conscripts (n=25,611) in order to identify factors that predispose for frequent consumption. A self-report of drinking frequencies, as well as socio-demographic and psychosocial variables, was collected at psychiatric baseline screening. Based on univariate analyses, relevant variables were included in a multivariate multinomial logistic regression model. Six percent were abstainers, 15% reported rarely drinking, 53% occasional drinking, 24% regular drinking and 2% daily drinking. Except for substance use, most associations followed a “J”-shaped curve across the categories of alcohol frequency. Abstinence and frequent drinking can be perceived as deviations from the social norm. Both behaviors are associated with more psychosocial stressors and might be therefore special targets for further studies and new prevention programs.

## 1. Introduction

The misuse of alcohol represents a serious problem, which affects all sections of society in most parts of the world. Ezzati and colleagues [1] estimated that 58 million disability adjusted life years (DALYs) are lost worldwide due to the misuse of alcohol (representing 4.0% of all DALYs). From an epidemiological perspective the consumption of alcohol as well as smoking tobacco has been identified as the greatest preventable danger to public health [2]. However, due to a more hedonistic drinking style, i.e. using alcohol for its pleasurable effects, especially young people are threatened by the misuse of alcohol. Almost half of the Swiss male population (42.2%) between 15–24 years of age are so-called binge drinkers, defined as drinking five or more alcoholic drinks in two hours within a 30-day period [3]. Heavy and frequent drinking can lead to acute adverse health consequences (e.g. intoxications, road traffic accidents) and long-term effects such as hypertension [4] or mental disorders [5]. There is further empirical evidence that excessive drinking behavior in adolescence represents an important predictor for future alcohol abuse and/or addiction problems [6]. In 1998, the estimated social and economic burden of alcohol abuse in Switzerland amounted to 6.5 billion Swiss Francs [7].

In Switzerland, one of the few countries still practicing conscription and requiring compulsory military service, all Armed Forces conscripts go through an early screening of any potential alcohol problems, including risk and concomitant factors, as well as adverse implications. Detailed knowledge of this behavior is certainly an important matter for the Armed Forces. It is the Armed Forces’ obligation and responsibility to care for the physical and mental health of everyone serving during basic military training (BMT) followed by repetition courses or being sent on real missions. Alcohol misuse already represents a potentially important safety risk factor, especially for all those in responsible and demanding positions, and even more so when handling vehicles or weapons. Furthermore, evidence exists that higher stress levels are often experienced during military training or service, which can lead to more frequent and heavy alcohol consumption [8]. Particularly during BMT, military service is associated with higher stress levels (physical conditions, sleep deprivation), while habitual coping strategies, such as arbitrary social environments, are neither adequate nor useful. Assessing profiles of alcohol use and pre-existing risk factors may permit early diagnosis of alcohol misuse and offer suggestions for the design of early interventions and specific prevention campaigns. Thus, the cross-social and economic impact on the military and general health care systems might be reduced.

The objective of this study was to evaluate drinking frequencies self-reported by a sample of Swiss conscripts, as well as to draw up sample-specific characteristics associated with alcohol consumption. It specifically focused on associated factors that can be easily observed or assessed at pre-military capability examinations. Easily available data such as age, psychosocial problems, etc., might be used by examiners to identify persons with a clearly increased risk for elevated levels of alcohol consumption. Profiles of known risk factors could facilitate the early detection of individuals at greater risk for hazardous drinking behavior, and allow their triage to subsequent clinical examinations.

## 2. Methods

### 2.1. Sample and Procedure

Due to required conscription of every Swiss male between ages 18 and 22 years, they are obliged to undergo a pre-military examination for physical and mental fitness, independently of whether the individual will eventually serve in the Armed Forces. This procedure also includes a psychiatric examination with a pre-selection screening procedure via an electronic survey as a first step. The data investigated in this study are the psychiatric screening data collected on Swiss Armed Forces conscripts in 2005. A total of 28,125 conscripts and female volunteers entered recruitment, and hence completed the psychiatric screening questionnaire. After excluding 713 damaged or incomplete records, as well as removing the too-small number of female volunteers (n=171), 27,241 conscripts were left in the dataset. Further restrictions were made for subjects with suspected simulation tendencies (malingering; n=1,630) [9] - operationalized by the SCL-90-R malingering definition (t-transformed Positive Symptom Total Score – T_PST_ ≥ 70) [10], so finally, the results presented in this paper are based on data from 25,611 male conscripts with a mean age of 19.15 years (SD=0.84). Compared to the excluded data, the final sample is slightly younger than the excluded volunteers (mean age 19.16±0.84 vs. 19.43±1.16 years, p<0.001) and the malingerers (mean age 19.15±0.84 vs. 19.33±0.90 years, p<0.001).

This project of the Medical Department of the Swiss Armed Forces was undertaken in collaboration with the Centre for Disaster and Military Psychiatry at the University of Zurich. The project was cleared by the Zurich State Ethical Committee (KEK) to fulfill all legal and data privacy protection requirements. All screening sessions were introduced and supervised by military test psychologists.

### 2.2. Materials

The psychiatric screening survey consisted of 291 questions covering the primary facets of psychopathology, as well as various additional psychosocial and behavioral aspects of mental health. It includes questions about alcohol consumption as an important correlate of mental and social wellbeing. One of these measures asked about drinking frequencies, i.e. “how frequent alcohol will be used?” The conscripts could specify their drinking behavior on five categories ranging from “never drink” through “daily drinking”. Table 1 shows in the upper section all response categories of drinking frequencies used in this study.

In order to search for possible risk and other associated factors of alcohol use, we investigated the following variables:

1. *Basic characteristics* such as age, social factors (having no steady partner, living alone – both dichotomized to true *versus* not true), career factors (no completed compulsory school, being unemployed - both dichotomized to “true” *versus* “not true”), as well as whether the individual was receiving a disability pension. For a better interpretability, all factors were negatively poled in the direction of higher risk for elevated alcohol consumption.
2. *Psychosocial stressors* were related to problems in the past 12 months in relevant areas of life, such as with *work* (conflicts at work, lost job), partnership (longer periods of separation from partner by professional or other reasons, left by partner), and school or training (high pressure to perform, arguments with teacher/employers or other students/trainees). All three problem-related variables were dichotomized into having at least one of these stressors versus none.
3. *Family situation* was assessed in terms of having parents with a migration background, being adopted, and/or having parents separated or divorced. All these items were dichotomized to “true” *versus* “not true”.
4. *Substance use* contained the three items: “Do you currently smoke tobacco?”, “Do you currently smoke cannabis?” and – herein after referred to as hard drugs – “Have you ever tried ecstasy, speed or amphetamines, cocaine, heroine or other opiates?”. Response options were “yes” *versus* “no”.
5. *Use of health care service* inquired about the average number of medical consultations per year. Four response categories were given: “never”, “once or twice”, “three to four”, and “Five or more times”.
6. *Own or family history of mental health* was characterized by suicidal behavior (dichotomized to having ever thought about or attempted suicide *versus* never), past or present psychological treatment (“yes” *versus* “no”), as well as alcohol abuse in family (at least one family member; “yes” *versus* “no”), other addictions in family (at least one family member; “yes” *versus* “no”) and psychiatric illness in at least one family member (“yes” *versus* “no”).

Moreover, the study included the Symptom-Checklist-90-Revised (SCL-90-R; [10]), a well-validated and widely used clinical measure for psychopathology. The SCL-90-R is a self-rating scale for mental health symptoms with 90 items, 83 of which reflect one somatic and eight psychiatric dimensions. The seven remaining items refer to disturbances in sleeping and eating behaviors for separate interpretations [10]. By means of the sub-scores, patterns of different symptom clusters can be obtained, or the total score can be used as an indicator of general psychiatric distress. In the current study, we used the Global Severity Index (GSI), which is equal to the SCL-90-R total score, as a measure of general psychopathology.

### 2.3. Statistical Analyses

Drinking frequencies of the study sample were analyzed in relation to the variables described above. Chi-Square Tests were used to examine differences in proportions, while mean differences in continuous variables were tested using one-way-ANOVAs. Variables that showed significant differences between categories of the outcome variable in univariate analyses were entered jointly into a multivariate logistic regression model. Frequency of alcohol consumption was measured by using an ordinally scaled variable what requires an ordered logistic regression as appropriate modeling strategy. However, testing the assumption of parallel regression revealed a violation of that assumption, which was to be expected due to such a large study population [11]. Alternatively, we used a multinomial model that ignores the ordinal structure of the response variable by treating its categories nominally scaled. All response categories were dummy-coded, i.e. comparing every higher category (“1”-coded) with the lowest category (“never drinking alcohol”; “0”-coded). For the final model the SCL-90-R was transformed into z-scores. Thus, sub-sample mean scores can be perceived as standard deviations from the total mean score. Odds ratio estimations (OR) and 95% confidence intervals were calculated for the strength of the association between each of the five categories of drinking frequency and the predictor variables adjusted for all other variables. For the sake of a better interpretability, the abstainer sub-sample served as reference category. Scale transformation (z-scores) of the GSI relied on the final study sample after any exclusion. All analyses were performed using STATA 9 for Macintosh [12].

## 3. Results and Discussion

### 3.1. Results

Examining the self-reported drinking frequencies, 6.3% conscripts described themselves as abstainers, 14.7% reported rare (max. five times per year) alcohol consumption, about half of the sample (53.1%) reported not exceeding one through five times per month (i.e., occasionally), whereas 23.8% reported drinking regularly (i.e., one through five times per week), and 2.2% daily.

Univariate analyses revealed that all independent variables significantly varied across the five outcome categories of drinking frequency, whereof most associations followed a “J”-shaped curve (“U”-shaped for age and disability pension). According to this, “never”, as well as “daily” (and “regular”) drinking, was more often related to psychosocial stressors (or associated with higher psychopathology, respectively) compared to “rare” and “occasional” drinking. For instance, lowest proportions of training- or school-related problems were found in occasional drinkers (15.7%), with an increasing tendency towards abstainers (21.9%) as well as daily drinkers (64.6%). These differences towards the extreme categories were almost higher for daily drinkers than for abstainers, except in those whose parents immigrated to Switzerland, which followed a converse “J”-shaped curve. Only substance use (tobacco, cannabis and hard drugs) was linearly related to the outcome (i.e. the higher the alcohol use, the more use of other substances was reported).

Health care utilization was related to drinking frequencies as follows: never seeing a doctor was negatively linear related to the drinking frequencies (ranged from 37.6% abstainers to 25.8% daily drinkers), bell-shaped in one or two doctor visits per year with a majority of occasional drinkers (54.2%), and linearly increasing in three through four service uses per year (ranged from 11.3% abstainers to 19.2% daily drinkers), up to an “J”-shaped curve in heavy service users (i.e., more than five times a year) with a majority of daily drinkers (14.8%) and a minority of occasional drinkers (4.3%). Table 1 displays detailed descriptive statistics for categorical and continuous variables, and their association with alcohol drinking frequencies, as well as difference test statistics.

Further evaluation of the data was performed using a multivariate multinomial logistic regression analysis, with all variables included – comparing elevated drinking frequencies with abstaining from drinking alcohol as the reference category (see Table 2). The results show that conscripts who drink occasionally or regularly are significantly younger than alcohol abstainers. Compared to never drinkers, regular drinkers were 23% more likely to have no partners, while daily drinkers had a 63% higher risk of living alone. Furthermore, overall drinking (versus abstinence) was less likely among those without compulsory school completion, without a job, those whose parents formerly immigrated to Switzerland, and those who receive disability pension or are presently or in the past involved in therapeutic treatment. These associations were not always significant for daily drinkers. Occasional or regular drinkers were half as likely to have been adopted as abstainers, and further, regular drinkers had a 19% lower likelihood of having divorced or separated parents. Daily drinkers, as well as occasional and daily drinkers, were up to 50% more likely to have experienced partner-related problems. Conscripts with school- or training-related problems were less likely to drink rarely to regularly than abstainers, but had an 85% higher risk of drinking daily. Furthermore, the results showed clearly that the higher the alcohol consumption rate was, the higher was the risk for tobacco smoking and/or cannabis use. For example, daily drinkers were more than 5 times as likely smoking tobacco and more than 6 times more often using cannabis than abstainers. Further, regular and daily drinkers tended to more frequently use hard drugs and to exhibit more often suicidal behavior than abstainers. Alcohol abuse or other addictions in the family were not respectively just weakly associated with drinking frequencies, while regular drinkers were 28% more likely to have a psychiatric illness in their family than abstainers. Between health care utilization and drinking frequencies, there was a significant and increasing association, i.e. conscripts with increasing alcohol-drinking frequencies (compared to abstainers) had higher odds of frequently using health care services. No significant associations were found for daily drinkers and their health care utilization, and between overall drinking and using health care services five times or more per year. Higher risks for mental pathology were found for regular and daily drinkers.

### 3.2. Discussion

This study analyzed alcohol-drinking frequencies in a sample of young Swiss male conscripts, and aimed to further investigate possible factors that may indicate higher consumption rates. Our analyses accessed a sample of 25,611 subjects aged 18–22.

The prevalence of male abstainers in Switzerland (14.2% in 2002) is high compared to other European countries, but low when compared to worldwide numbers [7]. In the present sample only 6.3% were abstainers, while 14.7% reported rarely drinking (max. five times per year), more than a half (53.1%) up to five times monthly, 23.8% up to five times weekly and only 2.2% reported daily consumption. In a study investigating approximately 46,000 Swedish conscripts, similar rates were found, at least for abstainers (5.9% compared to 8.0% in all Swedish males) and daily consumers (2.4%) [7,13]. Such numbers indicate a higher risk for this special age cohort, since Swiss conscripts are likewise representative of the healthy young males in Switzerland.

These drinking levels cannot be attributed to conditions within the Swiss Armed Forces, since the screening was carried out at recruitment and during the pre-military health examination. However, changing life circumstances during and following military service with elevated stress levels may result in increased alcohol consumption. Ong and Joseph [8] reviewed studies comparing military and non-military samples, wherein the first exceeded the latter by almost twice as high rates of heavy alcohol use. Having such a potential shift of drinking behavior in mind, it shows how important that not only screening and monitoring of individuals at risk might be, but also how military service conditions urgently need to generally limit and reduce the possibilities and occasions of alcohol consumption.

Comparing the drinking frequencies between subgroups reveals that frequent drinking was more prevalent in single males and those who were not living with others, even after adjusting for all other variables. Described in the literature as the “marriage effect”, living in a partnership is protective against alcohol misuse [14], whereas discrepant drinking patterns strongly predict relational stress [15]. Conscripts, who drink occasionally, regularly or daily, reported significantly more frequent partner-related problems than abstainers. Furthermore, for all the conscripts, abstainers were most likely to report no use of cigarettes, cannabis or hard drugs, with a linear increase across the categories of more frequent alcohol consumption; the increase in using drugs was only significant for daily drinkers when controlling for all other variables. That drinking is functionally related to other problematic behaviors like smoking and using drugs is well known, especially when highly deviating from the norm such as with daily drinking [16,17]. By contrast, low prevalence among abstainers can be interpreted as a consequence of their general way of life. Alcohol abstinence for whatever reasons is linked to generally less risky behavior [13].

A similar picture appeared for conscripts whose parents formerly immigrated to Switzerland. Increased drinking frequencies (*versus* abstinence) were paired with lower probabilities of having immigrant parents. One possible explanation was suggested by Amundsen and colleagues [18], in that adolescents with migration backgrounds will be socialized in an abstinence-promoting environment. Their drinking behavior merely reflects habits of immigrant populations that are influenced by different ideological and religious backgrounds.

In addition, and rather surprisingly, our abstainers were more likely to have not completed compulsory school, be unemployed and be receiving disability pension. Alcohol drinking conscripts (at least one drink per year) had significantly decreased odds ratios when compared to abstainers regarding these last three variables. Similar findings were discussed in a study on Swedish women, in that underlying reasons for not drinking often remain unclear [19].

Although not tested in this study, abstainers are a very heterogeneous group, including “sick quitters”, as well as former problem drinkers [20]. Johansson and colleagues [21,22] therefore concluded that unemployment in abstainers is most probable if they are ex-drinkers, otherwise abstinence does not significantly decrease employment probability. Despite the relatively young age, it might be assumed that conscripts probably had experience with alcohol use, with some of them even having early alcohol-related problems. Ancillary to this, there is some evidence that treatment for alcohol problems strongly predicts drinking abstinence [23]. Hence, it is not surprising that those conscripts who never drink alcohol have been in therapeutic treatment more often than others, after controlling for the remaining variables. Even the risk for school- or training-related problems is lower for rare to regular drinkers than abstainers, although again much higher for daily drinkers. Such “J”-shaped relationships may be the result of complex associations between psychosocial variables and other potential confounders [20]. Thus, both abstainers and heavy drinkers deviate from the social norm due to many potential factors. Univariate results supported a similar association between the frequency of alcohol consumption and mental health, in that rare and occasional drinkers reported fewer mental health symptoms than abstainers, whereas regular and daily drinker had the highest GSI scores. Although that relationship could not be reconfirmed for rare and occasional drinking in the full model, our results, however, support previous findings. O’Donnell and colleagues [24] found a nonlinear association between alcohol consumption and depressive symptoms, which followed a “U”-shaped trend. Similar associations were found for anxiety and other facets of mental illness [25]. The authors suggested that there must be a range of factors associated with mental health in abstainers, as well as with high-level drinking, that contributed significantly to elevated levels of psychiatric symptoms, and both appear to have deviated somehow from the social norm. This hypothesis finds further confirmation in a “J”-shaped association of suicidal behavior and drinking frequencies. Suicidal behavior was found to be more prevalent in abstainers, and even more so in high frequent drinkers, supported by multivariate results. Studies investigating risk factors for suicide attempts revealed that alcohol consumption, and psychiatric symptoms, are important agents of suicide behavior [26,27]. Current alcohol use was further associated with increasing health care use across the drinking frequency categories, whereas abstainers (and frequent drinkers) also slightly deviated from rare and occasional drinkers following such a “J”-curve. Unfortunately, this relationship did not hold up in multivariate testing. Therefore, the findings do not give a uniform picture, in that high-frequency service use was not related to alcohol use, and no significant relationships were found between daily drinking and health care utilization with simultaneous considerations of other variables.

All in all, it should be noted that, although significant, many of the differences between the categories of drinking frequency are rather small. However, no matter how small these differences are, they are important. Studying a complete age cohort of Swiss males represents a clear strength of this study, because of the large database the findings presented in this paper can be regarded as representative.

### 3.3. Limitations

Unquestionably, this study suffers from several limitations. The first and major limitation has to be the restricted informative value of causality. Alcohol use seems frequently to be associated with several potential confounders. But due to our cross-sectional design, it still remains unclear whether we are dealing with pre-existing, promoting risk factors, or on the contrary, with the consequences of alcohol use. A further limitation is given by the fact that alcohol consumption was summarized as frequency of alcohol use. However, such information provides a large sense of variability and thus a source of bias. Not every use of alcohol is hazardous. So might one person drink one or two drinks at lunch on every day of the week, whereas somebody else might drink the same accumulated amount on one occasion each weekend. Using the present measure to record drinking habits might overestimate the risk of alcohol use in the first mentioned case, while underestimating it in the latter. Our findings therefore provide rather rough estimations of alcohol use. Additional information about drinking quantities as well as frequencies of binge drinking may contribute to a more complex definition of drinking patterns. Another limitation concerns the validity of self-reported data as a potential source of biasing the results. Although it has shown that self-reports on drinking behavior may be affected by bias of underreporting [28]; most studies have found significant correlations between alcohol-related questions and alternative measures or sources of drinking behavior [29,30]. Self-reports may be considered as reliable and valid methods to measure alcohol consumption but should be interpreted with caution.

## 4. Conclusions

Our study provides further evidence that frequent use and probable misuse of alcohol is highly related to more problematic behaviors and other risk factors. However, it shows that abstinence is actually not linked to better mental health or less psychosocial problems than moderate drinking, but only to less risky behavior. As long as the underlying mechanisms for abstinence remain unclear, conclusions should be drawn with caution. Our findings only suggest that moderate alcohol use is an important part of social life, whereas more frequent as well as never drinking can somehow be considered as deviations from the norm. These results underscore not only the need for additional research examining factors of problematic alcohol use, but also indicate that further studies and prevention programs should be targeted to both ends of drinking continuum - abstinence and high-frequency.

## Figures and Tables

**Table 1.** Descriptive statistics and comparisons of personal data between categories of drinking frequency.

		Never n=1,581	Rarely (1–5 times/year) n=3,715	Occasionally (1–5 times per month) n=13,425	Regularly (1–5 times per week) n=6,023	Daily n=556	Test #
Basic characteristics	Age (mean=19.15, SD=0.84, range = 18–22; mean, SD)	19.25 (0.96)	19.21 (0.87)	19.10 (0.82)	19.16 (0.81)	19.34 (0.88)	p<0.001
	No steady partner (vs. steady partner; %)	59.9	59.0	59.9	63.8	59.6	p<0.001
	Living alone (vs. communal or familiar living; %)	3.9	3.2	3.0	4.0	11.2	p<0.001
	No completed compulsory school (vs. completed; %)	3.3	1.4	1.2	1.5	7.7	p<0.001
	Unemployed (vs. employed; %)	6.7	4.7	3.0	4.0	9.9	p<0.001
	Disability pension (vs. not receiving; %)	4.9	2.8	2.2	2.2	4.9	p<0.001
Psychosocial problems	Partner-related problems (vs. not; %)	14.2	13.9	16.3	22.9	34.0	p<0.001
	Work-related problems (vs. not; %)	20.4	18.9	18.7	27.1	48.0	p<0.001
	School- or training-related problems (vs. not; %)	21.9	17.4	15.7	30.1	64.6	p<0.001
Family situation	Parents immigrated to CH (vs. CH-resident parents; %)	32.4	26.3	17.4	16.6	21.0	p<0.001
	Adopted (vs. not; %)	2.2	1.4	0.9	1.0	3.5	p<0.001
	Parents separated or divorced (vs. not; %)	23.2	24.3	22.9	24.5	38.3	p<0.001
Substance use	Smoking (vs. non-smoking; %)	20.2	28.1	38.9	57.9	76.6	p<0.001
	Current use of cannabis (vs. not; %)	4.3	7.9	11.7	28.0	55.0	p<0.001
	Past or present use of hard drugs (vs. never tried; %)	2.8	3.4	4.0	10.2	35.1	p<0.001
Own and family history of mental health	Suicidal behavior (vs. never; %)	11.6	11.3	11.3	23.0	43.7	p<0.001
	Past or present therapeutic treatment (vs. never; %)	12.9	10.0	8.7	13.0	27.2	p<0.001
	Alcohol abuse in family (vs. not; %)	14.9	12.0	13.0	21.8	37.6	p<0.001
	Other addictions in family (vs. not; %)	14.4	13.3	12.7	20.3	33.8	p<0.001
	Psychiatric illness in family (vs. not; %)	24.4	22.7	23.9	36.3	52.2	p<0.001
Health Care Utilization	Never	37.6	34.2	29.7	25.8	25.8	p<0.001
	Once or twice per year	44.5	50.3	54.2	50.4	40.2	
	3 through 4 times per year	11.3	10.7	11.8	16.3	19.2	
	More than 5 times per year	6.6	4.8	4.3	7.5	14.8	
Mental pathology	SCL-90-R Global Severity Index GSI (mean=0.30, SD=0.31, range = 0–2.40; mean, SD)	0.27 (0.30)	0.25 (0.29)	0.25 (0.27)	0.41 (0.36)	0.61 (0.43)	p<0.001

# Chi-square test comparing proportions of categorical variables and one-way-analyses of variance (ANOVA) comparing means of continuous variables between the five categories of drinking frequency.

**Table 2.** Multivariate multinomial logistic regression analysis of drinking frequencies.

		Rarely (1–5 times/year) n=3,715	Occasionally (1–5 times/month) n=13,425	Regularly (1–5 times/week) n=6,023	Daily n=556
Basic characteristics	Age (unit: years)	0.95 (0.88–1.02)	**0.81 (0.76–0.86)**	**0.84 (0.78–0.90)**	0.96 (0.85–1.09)
	No steady partner	0.98 (0.86–1.11)	1.02 (0.91–1.14)	**1.23 (1.08–1.39)**	1.12 (0.90–1.40)
	Living alone	0.96 (0.68–1.35)	0.94 (0.69–1.28)	1.02 (0.73–1.41)	**1.63 (1.05–2.52)**
	No completed compulsory school	**0.50 (0.33–0.77)**	**0.44 (0.31–0.63)**	**0.46 (0.31–0.69)**	1.40 (0.84–2.35)
	Unemployed	**0.68 (0.52–0.89)**	**0.44 (0.34–0.56)**	**0.38 (0.29–0.51)**	**0.42 (0.28–0.65)**
	Disability pension	**0.64 (0.47–0.87)**	**0.51 (0.38–0.67)**	**0.44 (0.32–0.61)**	0.75 (0.45–1.26)
Psychosocial problems	Partner-related problems	1.06 (0.88–1.28)	**1.32 (1.12–1.56)**	**1.37 (1.15–1.63)**	**1.50 (1.15–1.96)**
	Work-related problems	1.00 (0.85–1.19)	0.96 (0.83–1.12)	0.90 (0.77–1.06)	1.12 (0.87–1.44)
	School- or training-related problems	**0.79 (0.66–0.94)**	**0.65 (0.56–0.76)**	**0.78 (0.66–0.93)**	**1.85 (1.41–2.42)**
Family situation	Parents immigrated to CH	**0.76 (0.66–0.87)**	**0.45 (0.40–0.51)**	**0.34 (0.30–0.40)**	**0.35 (0.27–0.45)**
	Adopted	0.75 (0.47–1.18)	**0.48 (0.32–0.73)**	**0.45 (0.28–0.73)**	1.10 (0.56–2.15)
	Parents separated or divorced	1.08 (0.93–1.25)	0.94 (0.82–1.08)	**0.81 (0.70–0.94)**	1.01 (0.80–1.29)
Substance use	Smoking	**1.53 (1.31–1.79)**	**2.61 (2.27–3.00)**	**4.64 (4.00–5.38)**	**5.39 (4.11–7.07)**
	Current use of cannabis	**1.92 (1.42–2.60)**	**2.67 (2.02–3.53)**	**4.30 (3.24–5.70)**	**6.24 (4.40–8.84)**
	Past or present use of hard drugs	1.13 (0.76–1.67)	1.25 (0.87–1.78)	**1.61 (1.12–2.30)**	**3.51 (2.33–5.29)**
Own and family history of mental health	Suicidal behavior	1.10 (0.88–1.37)	1.02 (0.84–1.25)	**1.23 (1.00–1.51)**	**1.63 (1.21–2.18)**
	Past or present therapeutic treatment	0.82 (0.66–1.01)	**0.71 (0.59–0.85)**	**0.61 (0.50–0.75)**	**0.68 (0.51–0.92)**
	Alcohol abuse in family	**0.81 (0.67–0.99)**	0.92 (0.77–1.09)	1.07 (0.89–1.28)	1.16 (0.88–1.53)
	Other addictions in family	1.02 (0.84–1.23)	0.88 (0.74–1.05)	0.92 (0.76–1.10)	0.92 (0.70–1.23)
	Psychiatric illness in family	1.02 (0.87–1.19)	1.11 (0.97–1.28)	**1.28 (1.10–1.49)**	1.20 (0.93–1.55)
Health Care Utilization	Once or twice per year vs. never	**1.22 (1.07–1.40)**	**1.48 (1.31–1.68)**	**1.47 (1.29–1.68)**	1.13 (0.87–1.46)
	3 through 4 times per year vs. never	1.06 (0.85–1.31)	**1.33 (1.10–1.61)**	**1.48 (1.20–1.82)**	1.09 (0.77–1.55)
	More than 5 times per year vs. never	0.87 (0.65–1.15)	0.95 (0.74–1.22)	1.15 (0.88–1.49)	1.41 (0.95–2.10)
Mental pathology	GSI (z-transformed - unit: standard deviations)	0.97 (0.89–1.06)	1.00 (0.92–1.08)	**1.37 (1.26–1.49)**	**1.38 (1.23–1.55)**

McFaddens R^2^=0.07

All values are odds ratio estimates with 95% confidence intervals

Reference were non-drinkers (abstainers; n=1,581)

Numbers printed in bold are significant at level p < 0.05
